# Revisiting Stem Cell-Based Clinical Trials for Ischemic Stroke

**DOI:** 10.3389/fnagi.2020.575990

**Published:** 2020-12-14

**Authors:** Joy Q. He, Eric S. Sussman, Gary K. Steinberg

**Affiliations:** ^1^Institute for Stem Cell Biology and Regenerative Medicine, Stanford University School of Medicine, Stanford, CA, United States; ^2^Department of Neurosurgery, Stanford University School of Medicine, Stanford, CA, United States; ^3^Department of Neurology and Neurological Sciences, Stanford University School of Medicine, Stanford, CA, United States; ^4^Stanford Stroke Center, Stanford Health Care, Stanford, CA, United States

**Keywords:** stem cells, clinical trials, ischemic stroke, transplantation, cell lineages

## Abstract

Stroke is the leading cause of serious long-term disability, significantly reducing mobility in almost half of the affected patients aged 65 years and older. There are currently no proven neurorestorative treatments for chronic stroke. To address the complex problem of restoring function in ischemic brain tissue, stem cell transplantation-based therapies have emerged as potential restorative therapies. Aligning with the major cell types found within the ischemic brain, stem-cell-based clinical trials for ischemic stroke have fallen under three broad cell lineages: hematopoietic, mesenchymal, and neural. In this review article, we will discuss the scientific rationale for transplanting cells from each of these lineages and provide an overview of published and ongoing trials using this framework.

## Introduction

Stroke is the leading cause of serious long-term disability, significantly reducing mobility in almost half of affected patients aged 65 years and older (Benjamin et al., 2017). Each year, 795,000 strokes occur in the US alone, and the annual economic impact of stroke is estimated at $33.9 billion. The current standard of care for ischemic stroke is acutely time-sensitive: administration of intravenous tPA is recommended within 4.5 h of stroke onset, and endovascular therapy in select patients within 24 h of stroke onset (Hacke et al., 2008; Berkhemer et al., 2015; Campbell et al., 2015; Goyal et al., 2015; Jovin et al., 2015; Saver et al., 2015, 2016; Albers et al., 2018; Nogueira et al., 2018). Beyond the acute period, there are currently no proven neurorestorative treatments for stroke. Despite numerous clinical trials, drug-based therapies, including selective serotonin reuptake inhibitors, amphetamines, and ion channel modulators, have not yielded significant benefits, perhaps due to the complex cellular disruption that occurs within damaged ischemic tissue (Chollet et al., 2011; Mead et al., 2015; Simpson et al., 2015; Yeo et al., 2017). Unlike other organs, the brain responds to ischemia by undergoing liquefactive necrosis, a process in which dead tissue liquefies and is cleared by brain resident phagocytes over months. This long-lasting inflammatory process results in substantial neurotoxicity, myelin degradation, and glial scarring, as well as releasing a host of neuroinflammatory mediators, including cytokines (TNF-a, IL-1b, IL-6, IL-20),chemokines (MCP-1, MIP1a), cellular adhesion molecules (immunoglobulins, cadherins, integrins), reactive oxygen species, and matrix metalloproteases (Lakhan et al., 2009; Ceulemans et al., 2010; Stonesifer et al., 2017; Chung et al., 2018; Zbesko et al., 2018). At the liquefactive core of the infarct, hematopoietic lineage (myeloid and lymphoid), mesenchymal lineage (endothelial and other stromal), and neural lineage (neurons, astrocytes, and oligodendrocytes) cells undergo extreme stress, interacting and dying within this inflammatory, acidic, and hypoxic milieu (Chung et al., 2018).To address the complex problem of restoring function in ischemic tissue, stem cell transplantation-based therapies have been investigated as potential restorative treatments for chronic stroke. Aligning with the major cell types found within the ischemic brain, stem-cell-based clinical trials for ischemic stroke have fallen under three broad cell lineages: hematopoietic, mesenchymal, and neural (Table 1). In this review, we will discuss the scientific rationale for transplanting cells from each of these lineages and provide an overview of published and ongoing trials using this framework.

**Table 1:** Lineage origin of transplanted cells in published clinical trials for ischemic stroke.

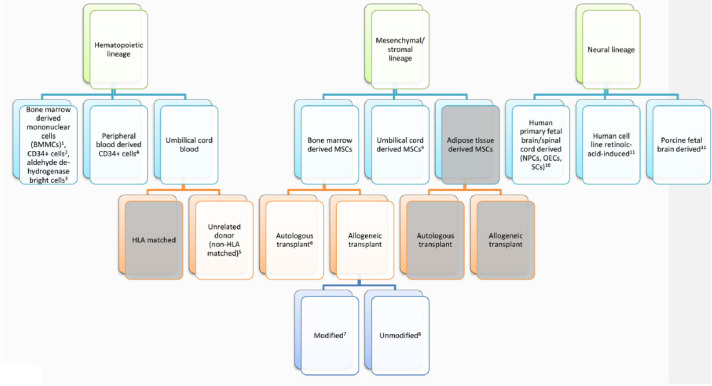

*^1^Suarez-Monteagudo et al. (2009), Barbosa da Fonseca et al. (2010), Savitz et al. (2011), Friedrich et al. (2012), Prasad et al. (2012), Prasad et al. (2014), Sharma et al. (2014) and Taguchi et al. (2015) ^2^Moniche et al. (2012) and Banerjee et al. (2014) ^3^Savitz et al. (2019) ^4^Chen et al. (2014) ^5^Laskowitz et al. (2018) ^6^Bang et al. (2005), Lee et al. (2010), Bhasin et al. (2011), Honmou et al. (2011) and Fang et al. (2019) ^7^Steinberg et al. (2016, 2018); ^8^Hess et al. (2017) and Levy et al. (2019) ^9^Chen et al. (2013) and Qiao et al. (2014) ^10^Chen et al. (2013), Qiao et al. (2014), Kalladka et al. (2016) and Muir et al. (2020) ^11^Kondziolka et al. (2000) and Kondziolka et al. (2005) ^12^Savitz et al. (2005). Shading, in pending clinical trials only (no results published)*.

## Hematopoietic Lineage

Hematopoietic stem cells (HSCs) at rest reside within the bone marrow niche but can be mobilized to the general blood circulation in response to stimulant factors such as granulocyte colony-stimulating factor (G-CSF). A majority of trials conducted using HSC lineage cells have relied on autologous bone marrow transplantation, favoring the lack of immunogenicity and ethical challenges to obtaining a reliable source of hematopoietic cells. In these studies, bone marrow was obtained from the patient and purified, either by density gradient alone or combined with immunosorting to obtain the cell population for transplantation. The cell surface marker CD34 characterizes a population enriched for HSCs, and the proportion of CD34+ cells found in the peripheral blood immediately after stroke has been found to directly correlate with functional recovery (Dunac et al., 2007).

### Bone Marrow Derived Cells

#### Bone Marrow Mononuclear Cell (BM-MNC) Transplantation

##### Early Phase I Trials (2009–2012)

The first Phase I trials of bone-marrow-derived cells demonstrated unequivocally that BM-MNCs could safely be transplanted in stroke patients at varying time points after stroke onset, and *via* various routes of administration. Suarez-Monteagudo et al. (2009) implanted 14–55 million autologous BM-MNCs by stereotactic intralesional injection in five chronic stroke patients at least 1 year and no more than 10 years post-stroke onset. This trial found that intracranially-injected autologous BM-MNCs were well-tolerated and safe, with several patients reporting long-term neuropsychiatric improvements. Targeting the subacute phase of ischemic stroke, Barbosa da Fonseca et al. (2010) infused 125–500 million autologous BM-MNCs in six patients *via* intra-arterial injection 8–12 weeks after stroke onset and reported no cases of neurologic worsening. In the acute setting, Savitz et al. (2011) determined that intravenous infusion of 70–100 million cells per kilogram weight of autologous BM-MNCs in 10 patients with acute stroke (24–72 h after onset) was both safe and feasible. Additionally, Prasad et al.’s (2012) research group intravenously infused 11 patients with 80 million autologous BM-MNCs (mean 0.92 × 10^6^ CD34+ cells) within 7–30 days of stroke onset and also confirmed the safety and feasibility of this treatment protocol. Friedrich et al. (2012) administered 50–600 million autologous BM-MNCs *via* intra-arterial (MCA) infusion to 20 patients within 3–10 days of stroke onset and found this method to be safe. Of note, while not known at the time, the brain biodistribution of intra-arterially and intravenously transplanted BM-MNCs was eventually determined to be comparable in a separate clinical trial completed a year later (Rosado-de-Castro et al., 2013). In both cases, brain biodistribution was low compared to that of lung or spleen, or liver. A summary of different routes of administration and their relative advantages and disadvantages concerning reaching ischemic brain tissue have been included in Table 2.

**Table 2:** Routes of administration of completed stem cell trials for ischemic stroke.

Intravenous (IV)	Intra-arterial (IA)	Intracranial (IC)	Intrathecal	Intranasal
Bang et al. (2005); Lee et al. (2010); Bhasin et al. (2011); Honmou et al. (2011); Savitz et al. (2011); Chen et al. (2013); Prasad et al. (2014); Qiao et al. (2014); Taguchi et al. (2015); Hess et al. (2017); Laskowitz et al. (2018); Fang et al. (2019) and Levy et al. (2019)	Barbosa da Fonseca et al. (2010); Friedrich et al. (2012); Moniche et al. (2012); Banerjee et al. (2014) and Savitz et al. (2019)	Kondziolka et al. (2000, 2005); Savitz et al. (2005); Suarez-Monteagudo et al. (2009); Chen et al. (2013, 2014); Kalladka et al. (2016); Steinberg et al. (2016, 2018) and Muir et al. (2020)	Chen et al. (2013) and Sharma et al. (2014)	
**Systemic administration** Advantage: • Simple procedure/ease of delivery Disadvantages: • Poor biodistribution to the brain: 0.9% after 24 h (Rosado-de-Castro et al., 2013) • Reperfusion of lesion after cell delivery not confirmed	**Systemic administration** Advantage: • When infused into the internal carotid or middle cerebral arteries, cell delivery to the brain occurs before other organs Disadvantages: • Reperfusion of lesion after cell delivery not confirmed • Lowest biodistribution to the brain overall: 0.6% after 24 h (Rosado-de-Castro et al., 2013) • Potential for complications and/or thrombosis	**Local administration** Advantage: • Greatest cell retention at lesion site compared to intra-arterial or intravenous: in mouse studies, 60% IC (compared to ∼1.5% IV) after 3 days (Barish et al., 2017) Disadvantages: • Uneven cell distribution throughout the lesion (Li et al., 2000; Giraldi-Guimardes et al., 2009) • An invasive procedure, requiring sedation and stereotactic localization	**Local administration** Advantages: • Direct access to CSF circulation throughout the brain and spinal cord • Less complex procedure than intracranial Disadvantage: • The mechanism of cellular migration from CSF space into brain parenchyma remains unknown	**Local administration** Advantages: • Enhanced blood-brain barrier permeability • Ease of delivery Disadvantage: • Unproven: clinical trials are ongoing and no data has been published to date

*Shading = in pending clinical trials only (no results published)*.

In the context of intracranial cell administration, transplantation into peri-ischemic vs. directly lesioned areas was extensively investigated in preclinical stroke models. The lesioned area was determined to be a poor injection target site due to unstable vascular supply and a highly inflammatory microenvironment. Moreover, the lesioned area was shown to eventually become a non-functional, fluid-filled cyst, suggesting that post-stroke recovery would be due to changes at the periphery of the lesion, not the cystic core (Veizovic et al., 2001; Modo et al., 2002; Smith et al., 2012). Of the clinical trials that utilized intracranial administration, only the porcine neural cell transplantation study conducted by Savitz et al. (2005) injected directly into the infarct. The study, discussed in a later section of this review, was terminated by the FDA after two of the five patients developed adverse events including cortical vein occlusion, complex partial seizures, and ring-enhancing lesions.

##### Phase I/II, Phase II Trials (2014–2015)

As Phase I/II trials progressed, the safety of these treatment protocols was consistently demonstrated, but BM-MNC administration was not found to significantly improve neurologic outcomes in transplant cohorts. In a follow-up to their initial Phase I trial, Prasad et al. (2014) performed a Phase II randomized study in which 120 patients received either 280 million autologous BM-MNCs or placebo intravenously within 7–30 days of stroke onset; this study yielded no clinical benefit for BM-MNC transplantation over placebo. Sharma et al.’s (2014) research group pursued a different route of administration, identifying 24 patients with chronic stroke (onset between 4 months to 10 years) to receive 1 million BM-MNCs per kilogram body weight intrathecally. Intrathecal administration proved safe, and functional improvement was noted in treated patients, however, there was no control group for comparison. In a phase I/IIa trial, Taguchi et al. (2015) intravenously administered either 250 million or 340 million cells of autologous BM-MNCs and found a trend toward improved neurologic outcomes and cerebral perfusion in the high dose group. Studies by Bhasin et al. (2016) additionally found that positive neurologic outcomes in patients given autologous BM-MNCs could be due to paracrine effects of secreted vascular endothelial growth factor (VEGF) and brain-derived neurotrophic factor (BDNF).

#### Sorted CD34+ From Bone Marrow

Hypothesizing that the hematopoietic stem cell-enriched CD34+ fraction of BM-MNCs contained the functional subset of cells responsible for repair during ischemic CNS injury, Moniche et al. (2012) conducted a Phase I/II study in which 160 million autologous CD34+ BM-MNCs were infused intra-arterially (MCA) into 10 patients within 5–9 days of stroke onset. The comparison group consisted of 10 subacute stroke patients who did not receive the intervention. In this trial, there was no clinical benefit at 180 days, but there was a statistically significant increase in b-NGF among the treated cohort (*p* < 0.02). Similarly, Banerjee et al.’s (2014) research group intra-arterially infused (*via* the MCA) 10 million autologous sorted CD34+ cells in five patients within 1–7 days of stroke onset, and found the intervention to be safe, and associated with a decrease in infarct size over time.

#### Sorted Aldehyde Dehydrogenase-Bright (ALDH-BR) From Bone Marrow

Aldehyde dehydrogenase (ALDH) was among the first markers used by immunologists to identify populations of human hematopoietic stem and progenitor cells in the 1990s. HSCs were found to express the highest levels of ALDH, while lymphocytes expressed the lowest (Kastan et al., 1990). Transplant of BM-derived ALDH-br cells was reported to improve functional recovery in limb ischemia and ischemic heart failure (Keller, 2009; Perin et al., 2011, 2012). Based on these findings, Savitz et al. (2019) transplanted up to eight million autologous ALDH-br BM-MNCs (dubbed autologous ALD-401 cells) *via* intracarotid infusion in 29 patients and compared this cohort to 19 control patients who received a sham procedure. Disappointingly, the study found no significant difference in primary or secondary efficacy measures between treatment and placebo groups.

### Mobilized Peripheral Blood Stem Cells

Shyu’s research group previously demonstrated peripheral blood-derived stem-cell (PBSC) transplant efficacy in treating chronic ischemia in rats (Shyu et al., 2006). To further evaluate the efficacy of this cell type in human subjects, Chen et al. (2014) conducted a Phase II trial in which autologous PBSCs were mobilized with G-CSF, and 3–8 million sorted CD34+ cells were stereotactically transplanted into 15 chronic stroke patients (stroke onset ranging from 6 months to 5 years prior). The authors noted improvement in multiple neurologic and functional outcome scores in the treated cohort.

### Umbilical Cord Blood Derived Cells

Allogeneic umbilical cord blood is an immunologically tolerant source of readily available cells. Unlike other sources of HSCs, HLA-matching is not required, and its safety and efficacy as a blood donor graft have been well established (Zhou et al., 2012). In this context, Laskowitz et al. (2018) conducted a Phase I study to establish the safety and feasibility of administering a single intravenous infusion of allogeneic (non-HLA matched) umbilical cord blood in stroke patients. The study included 10 patients treated between 3 and 9 days post-stroke onset, and noted an improvement in neurological and functional outcome, although there was no control group for comparison. A list of pending and ongoing clinical trials using cells of hematopoietic origin have been compiled in Table 3.

**Table 3:** Ongoing and pending unpublished hematopoietic lineage trials.

ClinicalTrials.gov Identifier	Study type	Cell type	Planned enrollment	Timing of delivery	Delivery route	Status
NCT01518231 “AHSCTIS” China	Phase I Randomized Open-label	CD34+ from peripheral blood	40	<12 months	IA	Recruiting/unknown
NCT00473057 Brazil	Phase I Non-Randomized Open-label	Autologous BM-MNC	12	<90 days	IA or IV	Completed 2011, no publications to date
NCT01832428 “BMACS” India	Phase I/II Non-Randomized Open-label	BM-MNC	50	Chronic	IT	Not currently recruiting/unknown
NCT02290483 also identified by NCT02178657 Spain	Phase II Randomized Open-label	BM-MNC (2 × 10^6^ or 5 × 10^6^ cells/kg)	76	<7 days	IA	Recruiting/paper published on miRNAs from the trial but no study results to date
NCT02795052 “NEST” USA	Phase n/a Non-Randomized Open-label	Autologous BMSC (intranasal for hypothesized blood-brain barrier penetration)	300	>6 months	IV or intranasal	Recruiting

## Mesenchymal Lineage

Mesenchymal stem cells (MSCs) are stromal cell precursors to cells of osteogenic, chondrogenic, and/or adipogenic lineages, and can be harvested and expanded from a variety of tissue types, including bone marrow (BM-MSCs, a cell population distinct from hematopoietic origin BM-MNCs), adipose tissue (adipose-derived MSCs, or AD-MSCs), and umbilical cord (UB-MSCs). This versatility, combined with low immunogenicity due to low expression of human leukocyte antigens, makes MSCs excellent candidates for allogeneic stem cell transplants (Le Blanc et al., 2003; Klyushnenkova et al., 2005). Unlike bone marrow-derived hematopoietic cells, MSCs from a single source can be expanded for transplant into many individuals, providing both standardization and scalability for large clinical studies.

BM-MSCs have been shown to cross the blood-brain barrier and improve functional recovery after acute ischemic stroke in animal models (Chen et al., 2001; Lee et al., 2016), likely due to a paracrine effect by secreting neurotrophic, mitogenic, and angiogenic factors, including VEGF, BDNF, nerve growth factor, basic fibroblast growth factor, and insulin-like growth factor 1 (Eckert et al., 2013; Shichinohe et al., 2015; Stonesifer et al., 2017). One hypothesized mode of delivery for these factors is *via* the secretion of membrane fragments (extracellular vesicles, EVs) from transplanted cells (Bang and Kim, 2019; Surugiu et al., 2019). Recent preclinical data in organoids suggest that EVs alone may be sufficient to significantly decrease injury in a hypoxia-starvation model of injury, opening the possibility for future early phase clinical trials of EV delivery for ischemic stroke (Zheng et al., 2020).

Additionally, AD-MSC transplants have also demonstrated success in experimental ischemic stroke models, in which animals treated with AD-MSCs demonstrated increased expression of BDNF and enhanced nerve regeneration, and simultaneously reduced expression of pro-apoptotic proteins such as BCL-2 and BAX within the ischemic lesion (Li et al., 2016). Multiple preclinical studies of UB-MSCs showed that transplanted cells quickly homed to the site of injury in rat ischemia models and resulted in improved long-term neurologic outcomes (Zhang et al., 2017; Wu et al., 2018). These results were also most likely due to paracrine effects, as transplanted cells did not persist long term.

### Bone Marrow Derived MSCs

#### Autologous Transplant

Bang et al. (2005) were the first to conduct autologous MSC transplants in stroke patients, to introduce cells with the potential to provide trophic support for neurogenesis and/or neuromodulatory effects—functions that are limited with hematopoietic cells. This group conducted a Phase I/II trial in which 100 million culture-expanded autologous BM-MSCs (grown in fetal bovine serum-containing media) were intravenously infused in five patients at 5–7 weeks post-stroke onset. Compared to a control cohort of 25 patients, treated patients demonstrated consistent neurologic improvement at 3, 6, and 12 months post-transplantation. Based on the success of this first study, the same group subsequently conducted a larger observer-blinded trial consisting of 52 patients (16 transplanted, 36 control; Lee et al., 2010). Due to the significant time required to expand MSCs in culture, the investigators opted to decrease the time-to-transplant by administering an initial dose of 50 million MSCs, and subsequently administering an additional 50 million MSCs 2 weeks later (rather than a one-time treatment with 100 million MSCs as in the Phase I/II trial). Notably, clinical improvement was found to be correlated with intactness of the subventricular zone—a known neurogenic site—as demonstrated on diffusion-weighted MR imaging. The MSC-transplanted cohort exhibited a higher rate of functional recovery and lower mortality compared to the control cohort. The authors reported that the use of bovine serum proteins to expand MSCs in culture did not appear to result in zoonoses or other adverse effects.

Due to ongoing concern regarding the use of bovine serum, particularly in the context of infectious diseases such as Creutzfield-Jakob, a Phase I study using MSCs expanded with autologous human serum was also conducted and published by a different group (Honmou et al., 2011). Honmou et al.’s (2011) research group demonstrated the safety and feasibility of BM-MSCs expanded in culture with human autologous serum, injected intravenously in 12 patients. Human serum resulted in more rapid MSC expansion *in vitro* compared to bovine serum, and at 1-week post-transplant, the mean infarct volume, as measured on MRI, was reduced by approximately 20% in patients treated according to this protocol. Circumventing the issue of serum altogether, Bhasin et al.’s (2011) research group conducted a Phase I/II trial in which autologous MSCs expanded in culture under serum-free conditions were intravenously administered to 20 chronic stroke patients between 3 months and 2 years following stroke onset. While both the control (*n* = 20) and transplanted groups experienced statistically significant improvement, there was no statistically significant difference between the groups in terms of functional outcome. Bhasin et al. (2013) presented a follow-up study in 2013 in which mesenchymal cells were compared with hematopoietic/mononuclear cells. Again, stem cell transplantation was found to be safe and feasible, with no conclusive evidence for efficacy but a trend toward functional improvement. Their 2016 study, again transplanting BM-MNCs, was discussed previously.

In a separate Phase I/IIa study, Fang et al. (2019) compared BM-MSCs to autologous endothelial progenitor cells (EPCs), as well as to placebo in patients within 5 weeks of stroke onset. This study included 18 total patients: six transplanted with BM-MSC, six with EPCs, and six with saline (placebo). The BM-MSCs were expanded in fetal bovine serum culture, and EPCs were derived by seeding bone marrow mononuclear cells on fibronectin plates to select for adherent cells, and subsequently maintained in bovine serum culture with endothelial cell media. Two injections of 2.5 million cells per kilogram body weight were given approximately 1 week apart, and patients were followed for 4 years. While the trial was deemed safe, no functional or neurological difference was observed between the BM-MSC, EPC, and placebo groups.

#### Allogeneic Transplant

The fact that MSCs express low levels of human leukocyte antigen and are easily expanded in culture is a significant advantage over other cell-based therapies about stroke therapy (Le Blanc et al., 2003). These features allow for the development of large quantities of standardized single-source cells for allogeneic transplant and also mitigate many of the challenges of autologous cell therapy in terms of timing of treatment. This is of particular benefit in patients who are unable to provide autologous cells. Moreover, because MSCs may be cultured and expanded *in vitro* without sacrificing stemness potential (Reyes et al., 2001; Jiang et al., 2002), genetic modification of these cells prior to transplantation in order to enhance supportive properties has also opened new possibilities.

##### Unmodified Cell Transplants

In the largest MSC trial to date, Hess et al. (2017; MASTERS, Athersys) conducted a phase II randomized, double-blind, placebo-controlled dose-escalation trial of intravenous adult BM-MSCs for acute ischemic stroke at 33 centers across the US and UK. In this trial, patients were randomized to receive either 400 million or 1.2 billion allogeneic BM-MSCs (*n* = 65) or placebo (*n* = 61) within 24–48 h after stroke onset. The allogeneic BM-MSC product used in this study—MultiStem—was derived from two independent donors (Boozer et al., 2009). No dose-dependent toxicity was observed and the treatment was deemed safe, but the transplanted group and placebo groups exhibited no significant difference in terms of functional outcome at 90 days post-stroke. Of note, a *post hoc* analysis of those patients achieving an “Excellent Outcome” defined as mRS ≤1 and NIHSS ≤1 and Barthel ≥95, demonstrated a statistically significant benefit for all patients treated at 1 year (23.1% transplant vs. 8.2% placebo; *p* = 0.02) and for all patients treated within the originally planned time window of ≤36 h post-stroke the benefit at 1 year was even greater (29.0% transplant vs. 8.2% placebo; *p* < 0.01). This finding was encouraging enough for Athersys to initiate another Phase III prospective, randomized, placebo-controlled, double-blind trial treating patients between 18–36 h of stroke (MASTERS-2).

In a separate Phase I/II trial using allogeneic single-donor adult mesenchymal BM-MSCs (Levy et al., 2019; Stemedica) transplanted up to 1.5 million BM-MSCs per kg body weight intravenously in 38 patients with chronic stroke (>6 months post-stroke). The highest dose (1.5 million/kg) was found to be safe, and significant behavioral gains were observed. Excellent functional outcome (Barthel score >95) was reported in 35.5% of patients at 12 months post-transplant, compared to in 11.4% at baseline; however, there was no control group included in this study.

##### Modified MSC Transplants

SB623 is a BM-MSC line that has been transiently transfected with a plasmid containing the human Notch1 intracellular domain, which results in constitutive Notch1 expression. Importantly, the Notch1 plasmid is not replicated during mitosis and is therefore rapidly lost during cell division. In *in vitro* preclinical models, Notch1-modified MSCs promoted neural cell growth and rescued neural cell survival after ischemia by providing trophic support *via* the secreted extracellular matrix, promoting angiogenesis, and playing a protective, anti-inflammatory role (Aizman et al., 2009; Tate et al., 2010; Dao et al., 2011, 2013). Experimental stroke models transplanted with Notch-1-modified MSCs have demonstrated functional recovery and peri-infarct neuroprotection as measured by a reduction in cell loss. Despite these benefits, however, the SB623 cells themselves are short-lived *in vivo* (Yasuhara et al., 2009; Tajiri et al., 2013). This finding suggests that observed improvements are a result of supportive trophic activity rather than engraftment, and alleviates concerns over the challenges of achieving long-term allogeneic cell engraftment.

Based on this encouraging preclinical data, Steinberg et al. (2016) completed a Phase I/IIa trial in which 18 chronic stroke patients (6 months to 3 years post-stroke onset) received a stereotactic intracranial injection of either 2.5, 5, or 10 million allogeneic modified SB623 BM-MSCs (six patients per cohort). Each patient received five stereotactic image-guided injections of 20 μl each surrounding the infarct. Of note, the intracranial administration of SB623 was chosen because of the desire to prioritize trophic factor delivery by transplanted cells over cell engraftment (Bliss et al., 2010). In comparison to intra-arterial and intravenous delivery, the intracranial injection was shown to result in greater delivery of transplanted cells to the lesion, although cells are unequally distributed throughout (Rosado-de-Castro et al., 2013). Patients who received intracranial injections reported several treatment-emergent adverse events (TEAEs), including headache, nausea, and vomiting. It was determined that most, if not all, of the TEAEs, were due to the surgical procedure rather than the cell transplantation. No link between TEAE and cell dosage was identified, and all TEAEs recovered without sequelae. No antibody response to SB623 cells was observed, and a significant improvement in neurological function was noted after 3, 6 (the pre-determined efficacy endpoint), and 12 months. In a follow-up article detailing 2-year outcomes, the initial improvements were found to be stable at 2 years post-transplantation (Steinberg et al., 2018). Interestingly, the authors noted that the size of a transient T2-FLAIR signal (DWI negative) on MRI at an early time point (1–2 weeks post-transplant) was correlated with the degree of long-term functional improvements, and could be a possible indicator of functional transplant activity. A larger Phase IIb randomized, double-blind, placebo-controlled study, ACTIsSIMA, has been completed but the results are as yet unpublished. Additional pending clinical trials using cells of mesenchymal origin have been detailed in Table 4.

**Table 4:** Ongoing and pending unpublished mesenchymal lineage trials.

ClinicalTrials.gov Identifier	Study type	Cell type	Planned enrollment	Timing of delivery	Delivery route	Status
NCT01678534 “AMASCIS-01” Spain	Phase I/II Randomized Double-blinded	Allogeneic cultured single donor Adipose-derived MSCs	40	<14 days	IV	Completed—no results to date
NCT01716481 “STARTING-2” South Korea	Phase III Randomized Open-label	Autologous BM-derived MSCs, expanded in serum	60	<90 days	IV	Recruiting/ unknown
NCT00875654 “ISIS” France	Phase II Randomized Open-label	Autologous BM-derived MSCs	31	<6 weeks	IV	Completed, no results to date
NCT02448641 “ACTIsSIMA” USA	Phase II Randomized Double-blinded	BMMNC SB623 (cultured single donor modified BM-derived MSCs)	156	6 months–7.5 years	IC	Completed, no results to date
NCT03570450 “RESSTORE-1” European Union	Phase I Randomized Open-label	Allogeneic adipose-derived mesenchymal stem cells	15	<1 week	IV	Recruiting
NCT02813512 Taiwan	Phase I Non-Randomized Open-label	Autologous ADSCs	6	>6 months	IC	Completed 2018
NCT03356821 ”PASSIoN” Netherlands **Perinatal/neonatal stroke**	Phase I/II Non-Randomized Open-label	One dose of 50 × 10^6^ Allogeneic BM-MSCs	10	<1 week	Nasal	Not yet recruiting
NCT03545607 “MASTERS-2” USA	Phase III Randomized Double-blinded	1.2 × 10^6^ MultiStem (Allogeneic BM multipotent progenitors)	300	18–36 h	IV	Recruiting (see Hess et al., 2017 for MASTERS)
NCT02378974 S Korea	Phase I/II Randomized Double-blinded	Human Umbilical cord-derived Mesenchymal Stem Cells (“Cordstem-ST”)	19	<7 days	IV	Completed, results not yet published
NCT04063215 USA **chronic brain injury**	Phase I/II Non-Randomized Open-label	Autologous adipose-derived Mesenchymal Stem Cells (HOPE biosciences)	24	>6 months	IV	Recruiting

## Neural Lineage

Neural stem cells (NSCs) are multipotent cells that can differentiate into neurons, astrocytes, and oligodendrocytes. This native capacity to repopulate and support endogenous cell types within the brain has generated much interest in NSC transplantation for stroke. The subventricular zone of the lateral ventricle and the dentate gyrus of the hippocampus have been identified as neurogenic sites, and in murine models, NSC’s have been shown to migrate from these niches to promote neurogenesis and vascular remodeling in response to ischemic stroke (Zhang et al., 2014; Hao et al., 2015).

One of the greatest barriers to developing an NSC transplantation model is the challenge of harvesting cells for transplantation. Clinical trials utilizing NSCs have attempted to overcome this barrier by: (1) modifying a human teratoma cell line [teratomas and cancer lines were the only non-embryonic source of pluripotent human cells before the development of induced pluripotent stem cells (iPSCs)] to induce neuronal differentiation by introducing the morphogen retinoic acid; (2) using NSCs harvested from human fetal tissue and/or clonal lines derived from these cells; or (3) using cells of non-human origin (i.e., porcine fetal NSCs).

### Retinoic Acid-Induced Differentiated Tumor Cells

Kondziolka et al. (2000) were the first to conduct NSC transplants for chronic stroke. In a Phase I trial published in 2000, 12 patients received either two or six million human “LBS-Neurons” *via* intracranial injection between 6 months and 6 years post-stroke. These cells were derived by differentiating the NT2/D1 human cell line, originally derived from a lung metastasis of testicular embryonal carcinoma, into neurons using a 10 μM dose of retinoic acid. Before the discovery of iPSCs in 2007, NT2/D1 was among the lines widely used to represent human pluripotent cells and was shown to be capable of terminal differentiation into multiple cell types, including neurons and astrocytes (Bani-Yaghoub et al., 1999). Preclinical studies demonstrated that NT2/D1-derived neurons resulted in improved functional outcomes after ischemia when injected intracranially (Borlongan et al., 1998). In humans, treatment with NT2/D1-derived neurons was found to be safe and associated with significantly improved functional outcome at 6 months.

Subsequently, the same University of Pittsburg group, with the addition of Stanford University investigators, conducted a Phase II randomized observer-blinded trial in which 14 chronic stroke patients received an intracranial injection of either five or 10 million LBS-Neurons; four patients acted as nonsurgical controls. While the primary efficacy endpoint—improvement of European Stroke Scale score at 6 months — was not achieved, one of the prespecified secondary outcome measures (the Action Research Arm Test designed to measure gross hand-movement) improved significantly compared with controls and with baseline scores on the same test.

### Porcine

Given the concerns surrounding implanting cells derived from malignant human tumors, compounded with the ethical implications and difficulty of obtaining fetal tissue, Savitz et al. (2005) attempted a Phase I trial to xenotransplant up to 50 million fetal porcine cells in five patients with chronic stroke, 18 months to 10 years post-stroke onset. Before intracranial transplantation, the cells were treated with anti-MHC to prevent rejection. While two patients reported clinical improvement, one patient developed seizures, and one experienced a temporary worsening of motor symptoms. As a result, this trial was halted due to safety concerns.

### Human Primary Fetal Brain Derived

The human fetal brain represents a source of actively dividing NSCs that have demonstrated proven engraftment and functional capacity in both preclinical and clinical studies for disorders such as leukodystrophies (Uchida et al., 2000, 2012; Tamaki et al., 2002; Kelly et al., 2004; Gupta et al., 2012, 2019); however, fetal tissue is difficult to obtain in the US due to governmental policy. CTX0E03 is one such fetal tissue line, derived by a research group in the UK and is currently being studied in ischemic stroke in the PISCES clinical trials (ReNeuron). CTX0E03 cells are a clonally derived human fetal cortical cell line that was transfected with a single copy of c-mycERTAM, an immortalizing gene dependent on tamoxifen administration for function (Pollock et al., 2006). Despite c-myc’s role as a known oncogene, preclinical stroke models indicated that CTX0E03 cells were safe to transplant, and promoted behavioral recovery *via* enhanced neurogenesis and angiogenesis in a dose-dependent fashion after ischemia (Stroemer et al., 2009). It is unclear if CTX0E03 cells exhibit long-term engraftment in the brain (Hicks et al., 2013; Baker et al., 2019). The PISCES 1 clinical trial for ischemic stroke transplanted 2.5, 5, 10, or 20 million CTX0E03 cells intracranially in 11 patients who had experienced a stroke 6–24 months prior (Kalladka et al., 2016). The treatment was found to be safe, and treated patients exhibited improved neurologic outcomes on several scales. However, the initial study was limited to cisgender male patients due to concerns about the potential for estrogen to activate c-myc. The PISCES 2 open-label Phase 2 trial transplanted 20 million CTX0E03 cells into the putamen of 23 patients, 13 males, and 10 females, 2–13 months after subcortical ischemic stroke (individuals actively taking tamoxifen were excluded). While the primary endpoint [two patients improving two points in the Action Research Arm Test (ARAT) subtest 2 at 3 months] was not met, there were substantial improvements in this metric as well as the mRS and Barthel Index (Muir et al., 2020). A Phase III prospective, randomized, controlled, double-blinded study (PISCES 3) treating 130 patients 6–12 months post-stroke is currently ongoing in the US.

### Cotransplant Studies of Primary Fetal NSC and UB-MSCs

Given the significant trophic support provided by mesenchymal cells and the regenerative capacity of NSCs, combined engraftment of fetal-derived neural and cord-blood-derived mesenchymal lineage cells together has been investigated as a potential therapeutic strategy to improve and support stem cell engraftment. Chen et al. (2013) sought to establish the safety and feasibility, as well as the optimal route of cell administration, of a multiple cell type co-transplantation in a group of 10 patients. The investigators isolated three distinct neural cell types from a single fetal donor: (1) olfactory ensheathing cells (OECs) from the fetal olfactory bulb; (2) neural progenitor cells (NPCs) from the subependymal zone; and (3) Schwann cells (SCs) from the sciatic nerve. Cord blood from a separate donor was used to derive UB-MSCs, and all transplanted UB-MSCs were derived from a single cord. Chronic stroke patients who were 6 months to 20 years post-stroke onset were included, and were divided amongst five distinct treatment protocols: (1) OECs alone (intracranial, *n* = 2); (2) OECs + NPCs (intracranial, *n* = 2); (3) OECs + NPCs (intracranial) with a second dose of NPCs at a later time point (intrathecal, *n* = 4); (4) OECs + NPCs (intracranial) + later doses of NPCs (intrathecal) and UB-MSCs (intravenous, *n* = 1); and (5) OECs and NPCs (intracranial) + later doses of SC and NPCs (intrathecal) and UB-MSCs (intravenous, *n* = 1). Treatment was found to be safe and feasible in all cases, however, due to the variety of cell types and routes of administration evaluated, the authors concluded that the study was not sufficiently powered to conclude that co-transplantation is safe under all studied conditions. The authors also noted a trend towards functional benefits with intracranial injections, but not with intrathecal and intravenous cell administration.

Another Phase I study conducted by Qiao et al. (2014) involved co-transplantation of UB-MSCs with human fetal cells. Enrolled patients received either four intravenous doses of 0.5 × 10^6^ UB-MSC cells per kg body weight or one intravenous dose of 0.5 × 10^6^ UB-MSC cells per kg body weight followed by three intrathecal doses of 0.5 × 10^6^ UB-MSC cells per kg body weight and 6 × 10^6^ human fetal derived NPCs (Qiao et al., 2014). In total, the investigators treated six subacute to chronic stroke patients between 1 week and 2 years post-stroke onset. Two of these patients received UB-MSCs only. The trial demonstrated that co-transplantation was safe and feasible; no malignancies were observed from the use of multipotent fetal cells. Further, each treated patient experienced clinical improvement that was stable at 2 years post-transplant. A list of pending clinical trials using cells of neural origin has been compiled in Table 5.

**Table 5:** Ongoing and pending unpublished neural lineage trials.

ClinicalTrials.gov Identifier	Study type	Cell type	Planned enrollment	Timing of delivery	Delivery route	Status
NCT01327768 China	Phase I Randomized Single blinded (participant)	OECs from nasal mucosa (autologous)	6	6–60 months	IC	Recruiting/unknown
NCT03629275 “PISCES-III” USA	Phase II Randomized	CTX0E03 modified NSCs, 20 million	130	6–24 months	IC	Recruiting

## Conclusions

The regenerative properties of stem cells have brought cell-based transplantation studies into the spotlight as appealing therapies for otherwise recalcitrant disorders such as subacute and chronic ischemic stroke. In this review article, we have detailed the clinical trials to date, which have featured transplantation of various cell types, administered *via* a variety of routes and in a variety of doses, to treat ischemic stroke of varying chronicity. We have discussed the history of and scientific rationale for the different cell types transplanted, their routes of administration, and associated trial outcomes, and have provided snapshots of current ongoing trials. Additional studies are necessary to strengthen our understanding of the relationship between neural cells and their surrounding stromal, endothelial, and immune landscape in both the healthy state and in pathologic conditions. As discussed in this review, specific attention should be given to the paracrine mechanisms by which transplanted cells exert their therapeutic effect, especially in light of data that suggests that these benefits persist even after the clearance of the originally transplanted cell type.

Furthermore, promising preclinical studies require adequate support and prudent design to overcome the “translational roadblock,” a notable decrease in efficacy between preclinical studies and their clinical trial counterparts. The difference is thought to be due to a series of factors. First, a combination of publication bias and overstated efficacy in preclinical studies has led to overly optimistic preclinical data that fail to result in statistically meaningful clinical interventions (Dirnagl et al., 2009; Macleod et al., 2009). Poor translation to the clinic has also resulted from the differences in primary endpoints between animal models and trials, lengthening of time-to-treatment in the clinic compared to in animal models, nuances in translating dosage, and heterogeneity of patient characteristics such as age (Dirnagl et al., 2009; Hermann et al., 2019). Recent studies have shown patient age to be a significant prognostic factor, and have suggested that, for clinical improvement, the timing of interventions must be increased to account for increased age (Sandu et al., 2017). Additionally, the underpowered clinical trial design has also contributed significantly to the difficulty of translating otherwise promising preclinical interventions to the bedside (Dirnagl and Macleod, 2009; Schmidt-Pogoda et al., 2020). Judiciously guiding the development of future stem-cell-based clinical interventions, including those harnessing recent advances in cellular regeneration, trophic support, immunomodulation, and perhaps as-of-yet undiscovered mechanisms of repair, will be essential in achieving successful clinical trials of promising neurorestorative therapies.

## Author Contributions

ES and GS contributed to the conception of this review. JH and ES contributed to the design and organization of the material. JH and GS created and updated the tables. JH, ES, and GS wrote sections of the manuscript, contributed to manuscript revision, read, and approved the submitted version.

## Conflict of Interest

GS is a consultant for Qool Therapeutics, Peter Lazic US, NeuroSave, SanBio, Audaxion Therapeutics, Zeiss and Surgical Theater. The remaining authors declare that the research was conducted in the absence of any commercial or financial relationships that could be construed as a potential conflict of interest.
