# Corrigendum: Revisiting Stem Cell-Based Clinical Trials for Ischemic Stroke

**DOI:** 10.3389/fnagi.2022.875925

**Published:** 2022-03-10

**Authors:** Joy Q. He, Eric S. Sussman, Gary K. Steinberg

**Affiliations:** ^1^Institute for Stem Cell Biology and Regenerative Medicine, Stanford University School of Medicine, Stanford, CA, United States; ^2^Department of Neurosurgery, Stanford University School of Medicine, Stanford, CA, United States; ^3^Department of Neurology and Neurological Sciences, Stanford University School of Medicine, Stanford, CA, United States; ^4^Stanford Stroke Center, Stanford Health Care, Stanford, CA, United States

**Keywords:** stem cells, clinical trials, ischemic stroke, transplantation, cell lineages

In the original article, we neglected to include the funder National Institutes of Health (NIH), R01 NS058784 to Gary K. Steinberg.

The authors apologize for this error and state that this does not change the scientific conclusions of the article in any way. The original article has been updated.

## Publisher's Note

All claims expressed in this article are solely those of the authors and do not necessarily represent those of their affiliated organizations, or those of the publisher, the editors and the reviewers. Any product that may be evaluated in this article, or claim that may be made by its manufacturer, is not guaranteed or endorsed by the publisher.

